# EGF-Induced EMT and Invasiveness in Serous Borderline Ovarian Tumor Cells: A Possible Step in the Transition to Low-Grade Serous Carcinoma Cells?

**DOI:** 10.1371/journal.pone.0034071

**Published:** 2012-03-30

**Authors:** Jung-Chien Cheng, Nelly Auersperg, Peter C. K. Leung

**Affiliations:** Department of Obstetrics and Gynecology, Child and Family Research Institute, University of British Columbia, Vancouver, British Columbia, Canada; University of Central Florida, United States of America

## Abstract

In high-grade ovarian cancer cultures, it has been shown that epidermal growth factor (EGF) induces cell invasion by activating an epithelial-mesenchymal transition (EMT). However, the effect of EGF on serous borderline ovarian tumors (SBOT) and low-grade serous carcinomas (LGC) cell invasion remains unknown. Here, we show that EGF receptor (EGFR) was expressed, that EGF treatment increased cell migration and invasion in two cultured SBOT cell lines, SBOT3.1 and SV40 large T antigen-infected SBOT cells (SBOT4-LT), and in two cultured LGC cell lines, MPSC1 and SV40 LT/ST-immortalized LGC cells (ILGC). However, EGF induced down-regulation of E-cadherin and concurrent up-regulation of N-cadherin in SBOT cells but not in LGC cells. In SBOT cells, the expression of the transcriptional repressors of E-cadherin, Snail, Slug and ZEB1 were increased by EGF treatment. Treatment with EGF led to the activation of the downstream ERK1/2 and PI3K/Akt. The MEK1 inhibitor PD98059 diminished the EGF-induced cadherin switch and the up-regulation of Snail, Slug and ZEB1 and the EGF-mediated increase in SBOT cell migration and invasion. The PI3K inhibitor LY294002 had similar effects, but it could not block the EGF-induced up-regulation of N-cadherin and ZEB1. This study demonstrates that EGF induces SBOT cell migration and invasion by activating EMT, which involves the activation of the ERK1/2 and PI3K/Akt pathways and, subsequently, Snail, Slug and ZEB1 expression. Moreover, our results suggest that there are EMT-independent mechanisms that mediate the EGF-induced LGC cell migration and invasion.

## Introduction

The epithelial-mesenchymal transition (EMT) is a highly conserved biological process during which there are multiple biochemical changes. This process results in the conversion of polarized, immotile epithelial cells into mesenchymal cells with a motile phenotype. This important process was initially recognized during critical phases of embryonic development, and recently, it has been shown that EMT is involved in promoting cancer cell invasion and metastasis [1].

A defining feature of EMT is a reduction in E-cadherin levels and a concomitant induction of N-cadherin [2]. Loss of E-cadherin expression is mainly due to an up-regulation of Snail, Slug, Twist, ZEB1 and other transcription factors that repress E-cadherin [3]. There is increasing evidence indicating that EMT is stimulated by signals from the tumor microenvironment, including a variety of growth factors and cytokines. In addition, EMT has been shown to be regulated by a series of intracellular signaling networks, including ERK1/2, PI3K/Akt, Smads, RhoB and β-catenin [4].

Epithelial ovarian cancer is the fifth leading cause of cancer-related deaths among women in developed countries. Most deaths from ovarian cancer are due to metastases that are resistant to conventional therapies. The epithelial growth factor receptor (EGFR) family consists of four members, EGFR (HER1), ErbB2 (HER2), ErbB3 (HER3), and ErbB4 (HER4), and has been shown to play an important role in metastasis and tumorigenesis in many types of human cancers [5], [6]. Amplifications and overexpression of the EGFR family have been reported in high-grade ovarian cancer and are associated with more aggressive clinical behavior and a poor prognosis [7], [8]. It has been shown that EGF can induce EMT in ovarian surface epithelium (OSE) and ovarian cancer cells, suggesting that EGF may be involved in ovarian cancer pathogenesis and metastasis [9], [10]. Ovarian cancer cells with low E-cadherin expression are more invasive, and the absence of E-cadherin expression in ovarian cancers is predictive of poor survival [11], [12]. Serous borderline ovarian tumors (SBOT) are non-invasive and are considered to be distinct entities that give rise to invasive low-grade serous carcinomas (LGC), which have a relatively poor prognosis when compared to SBOT and are unrelated to high-grade serous carcinomas [13]. Studies using clinical samples have shown that EGFR is expressed in borderline ovarian tumors [7], [14]. Although the function of EGFR signaling in cultured ovarian cancer cells has been studied, its function in the borderline tumors and in LGC is still unknown due to the lack of a suitable *in vitro* model. We recently established an *in vitro* culture system with human SBOT cells. Cultured SBOT cells grow slowly, are essentially non-invasive and exhibit limited motility. These characteristics resemble the cells' behavior *in vivo* [15]. Our recent study showed that p53 regulates the transition of SBOT cells from non-invasive to invasive ovarian carcinomas by activating the PI3K/Akt pathway and decreasing the expression of E-cadherin, indicating that EMT is a critical process for the regulation of SBOT cell invasion [16], [17].

In this study, we report for the first time that the EGFR is expressed in two cultured SBOT cell lines, SBOT3.1 and SBOT4-LT, and in two LGC-derived cell lines, MPSC1 and ILGC cells, and that EGF treatment induces cell migration and invasion in all cell lines. Interestingly, EGF only induces the cadherin switch in SBOT cells, which leads to SBOT cell migration and invasion. We also show that the underlying mechanisms involve the activation of the ERK1/2 and PI3K/Akt pathways. The information derived from this study provides critical insight into the role of EGFR activation in the down-regulation of E-cadherin, which plays a key role in increasing SBOT cell migration and invasion.

## Materials and Methods

### Cell culture

The SBOT3.1 [15], SV40 LT-infected SBOT (SBOT4-LT) [16] and SV40 LT/ST immortalized LGC (ILGC) [18] cell lines were established in our laboratory. SBOT and ILGC cells were grown in a 1∶1 (v/v) mixture of M199/MCDB105 medium (Sigma, Oakville, ON) supplemented with 10% fetal bovine serum (FBS, Hyclone Laboratories Inc., Logan, UT). The MPSC1 cell line, which was established from a LGC (provided by Dr. Ie-Ming Shih, Department of Pathology, Johns Hopkins Medical Institutions, Baltimore, MD), was maintained in RPMI 1640 (Invitrogen, Burlington, ON) supplemented with 10% FBS [19]. Cultures were maintained at 37°C in a humidified atmosphere of 5% CO_2_ in air.

### Antibodies and reagents

Polyclonal anti-EGFR and anti-β-actin antibodies were obtained from Santa Cruz Biotechnology (Santa Cruz, CA). The monoclonal anti-E-cadherin and anti-N-cadherin antibodies were obtained from BD Biosciences (Mississauga, ON). Monoclonal anti-phospho-EGFR (Tyr1173), anti-phospho-ERK1/2 (Thr202/Tyr204) anti-ZEB1 and anti-HER2 antibodies and polyclonal anti-ERK1/2, anti-phospho-p38 MAPK (Thr180/Tyr182), anti-p38 MAPK, anti-phospho-Akt (Ser473) and anti-Akt antibodies were obtained from Cell Signaling Technology (Danvers, MA). Polyclonal anti-Snail and anti-Slug antibodies were obtained from Abgent (San Diego, CA). Horseradish peroxidase-conjugated goat anti-mouse IgG and goat anti-rabbit IgG were obtained from Bio-Rad Laboratories (Hercules, CA). Horseradish peroxidase-conjugated donkey anti-goat IgG was obtained from Santa Cruz Biotechnology. Human epidermal growth factor (EGF), AG1478, SB203580 and LY294002 were obtained from Sigma. PD98059 was obtained from Calbiochem (San Diego, CA).

### Treatment methods

In the migration and invasion assays, cells with 80% confluence or cells treated with siRNA were treated with EGF for 24 (migration) and 48 (invasion) hr, respectively. After EGF treatment, cells were trypsinized and seeded into transwell inserts. For the general EGF treatment experiments, cells were cultured until 80% confluent and treated with 50 ng/ml EGF. The effect of EGF on the mRNA levels of E-cadherin, N-cadherin, Snail, Slug, Twist and ZEB1 were examined after 24 hr EGF treatment. The effect of EGF on the protein levels of those molecules were examined after 48 hr EGF treatment. The levels of specific mRNA and protein were examined by RT-qPCR and western blot, respectively. To knockdown EGFR, the cells were cultured until 60% confluent and then transfected with ON-TARGET*plus* SMART*pool* EGFR (50 nM) siRNA (Dharmacon Research, Inc., Lafayette, CO) using Lipofectamine RNAiMAX (Invitrogen) for 48 hr. The siCONTROL NON-TARGETING*pool* siRNA (Dharmacon) was used as the transfection control.

### Western blot

Cells were lysed in lysis buffer (Cell Signaling Technology), and protein concentrations were determined using a DC protein assay kit with BSA as the standard (Bio-Rad Laboratories). Equal amounts of protein were separated by SDS polyacrylamide gel electrophoresis and transferred to PVDF membranes. Following blocking with TBS containing 5% non-fat dry milk for 1 hr, membranes were incubated overnight at 4°C with primary antibodies, followed by incubation with HRP-conjugated secondary antibody. Immunoreactive bands were detected with enhanced chemiluminescent substrate. Membranes were stripped with stripping buffer and reprobed with anti-β-actin as a loading control. Band intensities were quantified using the Scion Image software and normalized to β-actin.

### Reverse transcription quantitative real-time PCR

Total RNA was extracted using TRIzol reagent (Invitrogen) according to the manufacturer's instructions. Reverse transcription was performed with 3 µg of RNA, random primers and M-MLV reverse transcriptase (Promega, Madison, WI). The primers used for SYBR Green reverse transcription-qPCR (RT-qPCR) were as follows: E-cadherin, 5′-ACA GCC CCG CCT TAT GAT T-3′ (sense) and 5′-TCG GAA CCG CTT CCT TCA-3′ (antisense); N-cadherin, 5′-GGA CAG TTC CTG AGG GAT CA-3′ (sense) and 5′-GGA TTG CCT TCC ATG TCT GT-3′ (antisense); Snail, 5′-CCC CAA TCG GAA GCC TAA CT-3′ (sense) and 5′-GCT GGA AGG TAA ACT CTG GAT TAG A-3′ (antisense); Slug, 5′-TTC GGA CCC ACA CAT TAC CT-3′ (sense) and 5′-GCA GTG AGG GCA AGA AAA AG-3′ (antisense); Twist, 5′-GGA GTC CGC AGT CTT ACG AG-3′ (sense) and 5′-TCT GGA GGA CCT GGT AGA GG-3′ (antisense); ZEB1, 5′- GCA CCT GAA GAG GAC CAG AG-3′ (sense) and 5′-TGC ATC TGG TGT TCC ATT TT-3′ (antisense); and GAPDH, 5′-GAG TCA ACG GAT TTG GTC GT-3′ (sense) and 5′-GAC AAG CTT CCC GTT CTC AG-3′ (antisense). RT-qPCR was performed on an Applied Biosystems 7300 Real-Time PCR System (Perkin-Elmer, Wellesley, MA) equipped with a 96-well optical reaction plate. All RT-qPCR experiments were run in triplicate, and a mean value was used for the determination of mRNA levels. Relative quantification of the mRNA levels was performed using the comparative Ct method with GAPDH as the reference gene and with the formula 2^−ΔΔCt^.

### Transwell migration and invasion assay

Migration and invasion assays were performed in Boyden chambers with minor modifications [20]. Cell culture inserts (24-well, pore size 8 µm; BD Biosciences, Mississauga, ON) were seeded with 1×10^5^ cells in 250 µL of medium with 0.1% FBS. Un-coated inserts were used for migration assays, whereas inserts pre-coated with growth-factor-reduced Matrigel (40 µl, 1 mg/ml, BD Biosciences) were used for invasion assays. Medium with 10% FBS (750 µl) was added to the lower chamber and served as a chemotactic agent. After 24 hr (migration) or 48 hr (invasion) incubation, non-migrating/invading cells were wiped from the upper side of the membrane. Cells that penetrated the membrane were fixed with cold methanol, and cell nuclei were stained with Hoechst 33258 and counted by epifluorescence microscopy with Northern Eclipse 6.0 software (Empix Imaging, Mississauga, ON). Triplicate inserts were used for each individual experiment, and five microscopic fields were counted per insert.

### Statistical analysis

Results are presented as the mean ± SEM of at least three independent experiments. Two-sample data were analyzed by Excel with the two-sample *t*-test assuming unequal variances. Multiple comparisons were analyzed by one-way ANOVA followed by Tukey's multiple comparison test using PRISM software. Significant differences were defined as *p*<0.05.

## Results

### Expression of E-cadherin, N-cadherin, EGFR and HER2 in cultured SBOT and LGC cells

**Figure 1:**
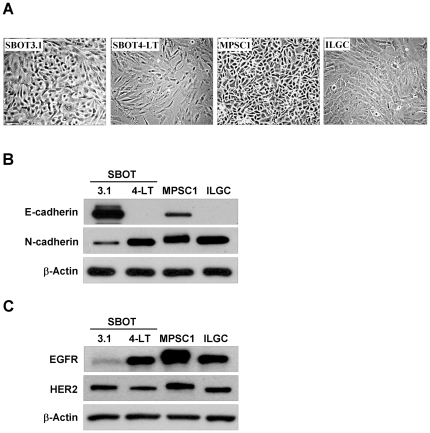
Expression of E-cadherin, N-cadherin, EGFR and HER2 in SBOT3.1, SBOT4-LT, MPSC1 and ILGC cells. A, The morphology of SBOT3.1, SBOT4-LT, MPSC1 and ILGC cells. The scale bar represents 100 µm. B, Endogenous protein levels of E-cadherin and N-cadherin were analyzed by western blot. C, Endogenous protein levels of EGFR and HER2 were analyzed by western blot.

Our recent studies showed that EMT is a critical process that contributes to the progression of non-invasive SBOT to invasive LGC [16], [17]. To confirm this result, we compared the basal expression levels of E-cadherin and N-cadherin in two SBOT lines, SBOT3.1 and SBOT4-LT, and two LGC-derived cell lines, MPSC1 and ILGC. SBOT3.1 cells grew slowly, whereas SBOT4-LT, MPSC1 and ILGC cells were grew faster. As shown in Figure 1A, SBOT3.1 and MPSC1 exhibited an epithelial morphology. With the introduction of SV40 LT or LT/ST, SBOT4-LT and ILGC exhibited a more atypical and scattered morphology. To compare the expression levels of E-cadherin and N-cadherin, cells were grown until they were fully confluent, and then the total proteins were collected. As shown in Figure 1B, the expression levels of E-cadherin were high in SBOT3.1 cells and low in MPSC1 cells, whereas the levels of E-cadherin were almost absent in SV40 immortalized SBOT4-LT and ILGC cells, which is consistent with our previous data showing that E-cadherin is down-regulated by the inhibition of p53 [16], [18]. These results indicate that MPSC1 cells are a more mesenchymal-like cell type compared to SBOT3.1 cells. To date, whether cultured SBOT and LGC cells express EGFR or HER2 remains unclear. As shown in Figure 1C, both SBOT and LGC cells expressed EGFR and HER2. The expression level of EGFR was lower in SBOT3.1 cells than in others, whereas all cell lines expressed similar levels of HER2.

### EGF treatment increases cell migration and invasion in SBOT and LGC cells

**Figure 2:**
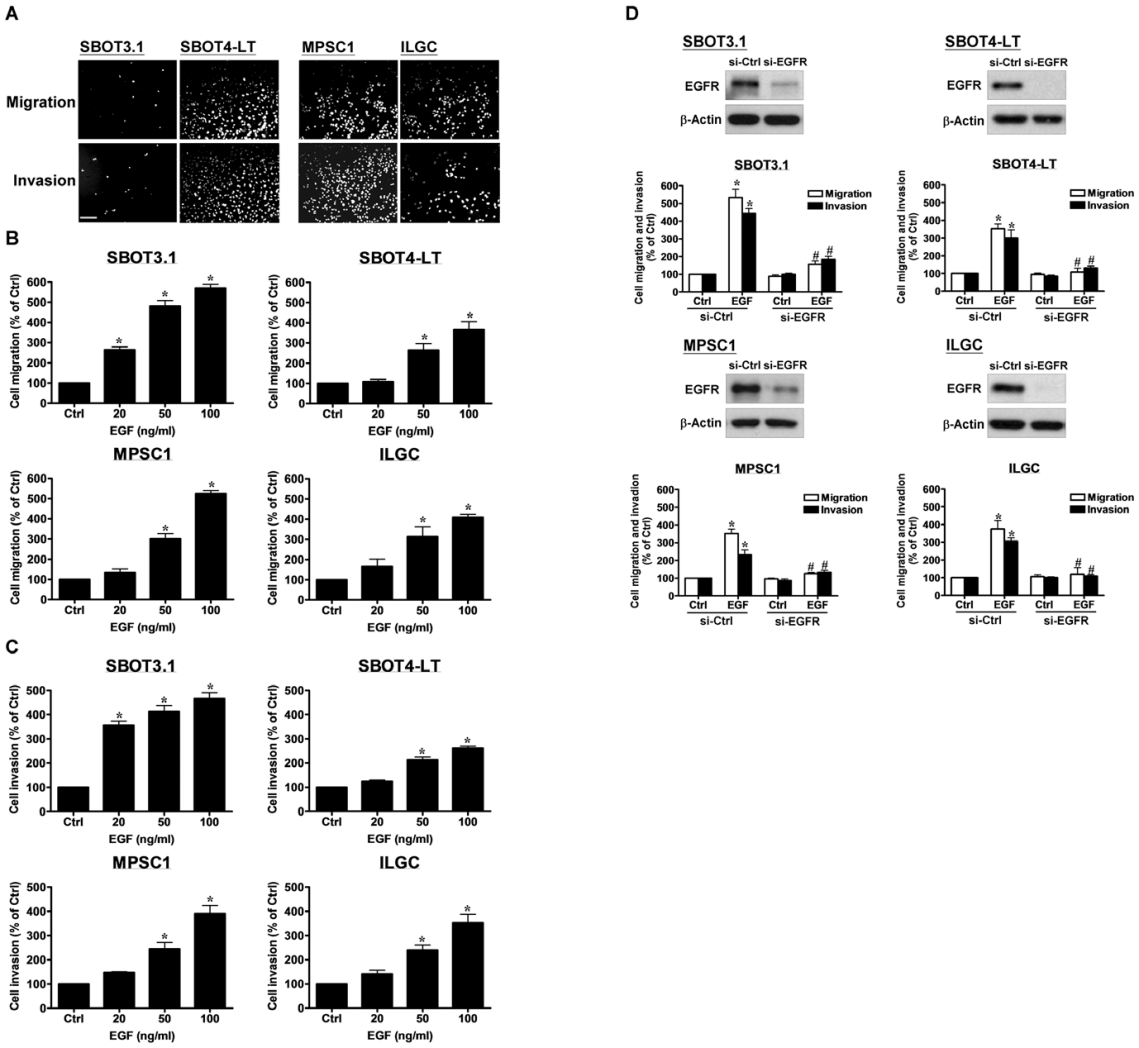
EGF induces cell migration and invasion in SBOT3.1, SBOT4-LT, MPSC1 and ILGC cells. A, The intrinsic migration and invasion of cells. B and C, Cells were treated with increasing doses of EGF (20, 50 and 100 ng/ml). D, Cells were transfected with control siRNA (si-Ctrl) or EGFR siRNA (si-EGFR) for 48 hr and then treated with 50 ng/ml EGF. After treatment cells were seeded into un-coated (migration) and Matrigel-coated (invasion) transwell inserts. After 24 hr (migration) and 48 hr (invasion) incubation, non-invading cells were wiped from the upper side of the filter and the nuclei of invading cells were stained with Hoechst 33258. Top panels show representative photos of the migration or invasion assay. Scale bar represents 200 µm. Bottom panels show summarized quantitative results which are expressed as the mean ± SEM of at least three independent experiments. Western blots show the knockdown of EGFR by EGFR siRNA. *p<0.05 compared with Ctrl. ^#^p<0.05 compared with EGF or EGF in si-Ctrl.

Transwell migration and invasion assays showed that SBOT3.1 cells were essentially non-motile and non-invasive, whereas SBOT4-LT, MPSC1 and ILGC cells were highly motile and invasive (Figure 2A). Interestingly, EGF treatment resulted in a significant increase in cell migration (Figure 2B) and invasion (Figure 2C) in a dose-dependent manner in all SBOT and LGC cell lines. To confirm the involvement of EGFR in EGF-induced cell invasion, EGFR-specific siRNA was used to knock down the endogenous EGFR. Western blot analysis showed that EGFR siRNA significantly knocked down the endogenous expression of EGFR. Moreover, EGFR siRNA abolished EGF-induced cell migration and invasion (Figure 2D). These results confirmed that EGFR is required for EGF-induced cell migration and invasion.

### EGF induces a down-regulation of E-cadherin and an up-regulation of N-cadherin in SBOT cells

**Figure 3:**
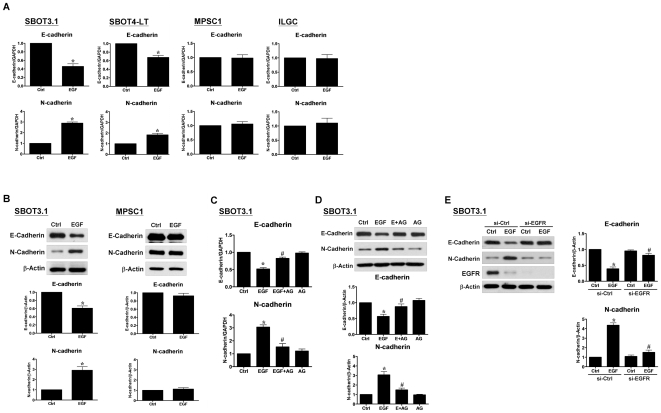
EGF induces cadherin switch in SBOT3.1 and SBOT4-LT cells, but not in MPSC1 and ILGC cells. A, Cells were treated with 50 ng/ml EGF for 24 hr. E-cadherin and N-cadherin mRNA levels were analyzed by RT-qPCR. B, Cells were treated with 50 ng/ml EGF for 48 hr. E-cadherin and N-cadherin protein levels were analyzed by western blot. C and D, SBOT3.1 cells were treated with the EGFR inhibitor, AG1478 (10 µM), in the presence or absence of 50 ng/ml EGF, and the levels of E-cadherin and N-cadherin mRNA (24 hr EGF treatment) and protein (48 hr EGF treatment) were analyzed. E, SBOT3.1 cells were transfected with control siRNA (si-Ctrl) or EGFR siRNA (si-EGFR) for 48 hr and then treated with 50 ng/ml EGF for 48 hr. The protein levels of E-cadherin and N-cadherin were analyzed by western blot. The results are expressed as the mean ± SEM of at least three independent experiments. *p<0.05 compared with time-matched Ctrl. ^#^p<0.05 compared with EGF or EGF in si-Ctrl.

A characteristic of EMT is a switch from E-cadherin to N-cadherin expression. In SBOT3.1 and SBOT4-LT cells, RT-qPCR analysis showed that EGF treatment down-regulated E-cadherin mRNA levels. Concurrently, N-cadherin mRNA levels increased with EGF treatment. Unexpectedly, EGF treatment did not alter the mRNA levels of E-cadherin or N-cadherin in MPSC1 and ILGC cells (Figure 3A). Similarly, western blot analysis performed on total cell lysates collected from cells treated with EGF for 48 hr showed that EGF down-regulated E-cadherin and up-regulated N-cadherin total protein levels in SBOT3.1 cells, but not in MPSC1 cells (Figure 3B). In addition, the effects of EGF on the mRNA and protein levels of E- and N-cadherin in SBOT3.1 cells were eliminated by treatment with the EGFR inhibitor, AG1478 (Figures 3C and D). Moreover, EGFR siRNA abolished the EGF-induced switch from E-cadherin to N-cadherin (Figure 3E). It has been shown that the binding of EGF to EGFR rapidly induces clustering and internalization of the ligand-receptor complexes, ultimately resulting in lysosomal degradation of both EGF and its receptor [21]. This process was supported by the data in Figure 3E, which shows that EGFR was down-regulated in SBOT3.1 cells in response to EGF treatment.

### EGF up-regulates Snail, Slug and ZEB1, but not Twist, in SBOT cells

**Figure 4:**
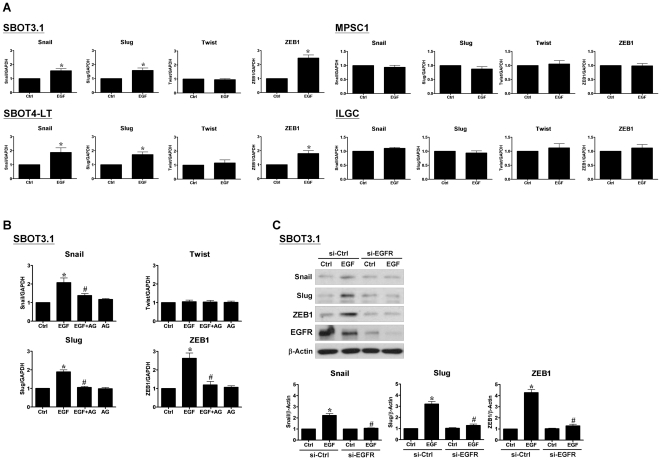
EGF induces Snail, Slug and ZEB1 expression in SBOT3.1 and SBOT4-LT cells, but not in MPSC1 and ILGC cells. A, Cells were treated with 50 ng/ml EGF for 24 hr, and the mRNA levels of Snail, Slug, Twist and ZEB1 were analyzed by RT-qPCR. B, SBOT3.1 cells were treated with AG1478 (10 µM) in the presence or absence of 50 ng/ml EGF for 24 hr, and mRNA levels were analyzed by RT-qPCR. C, SBOT3.1 cells were transfected with control siRNA (si-Ctrl) or EGFR siRNA (si-EGFR) for 48 hr and then treated with 50 ng/ml EGF for 48 hr. The protein levels of Snail, Slug and ZEB1 were analyzed by western blot. The results are expressed as the mean ± SEM of at least three independent experiments. *p<0.05 compared with Ctrl. ^#^p<0.05 compared with EGF or EGF in si-Ctrl.

To investigate whether EGF down-regulates E-cadherin expression by modulating the transcriptional regulation of E-cadherin, we used RT-qPCR to examine the mRNA levels of the E-cadherin transcriptional repressors Snail, Slug, Twist and ZEB1. Treatment with EGF significantly increased Snail, Slug and ZEB1 mRNA levels in SBOT3.1 and SBOT4-LT cells. However, EGF treatment did not alter the mRNA levels of Twist. In addition, the effects of EGF on these transcription factors were not detected in MPSC1 and ILGC cells, confirming that the E-cadherin is not transcriptionally regulated by EGF in LGC cells (Figure 4A). In addition, treatment with AG1478 abolished the effects of EGF on Snail, Slug and ZEB1 mRNA levels in SBOT3.1 cells (Figure 4B). Moreover, western blot analysis showed that EGFR siRNA abolished EGF-induced Snail, Slug and ZEB1 expression in SBOT3.1 cells (Figure 4C).

### Activation of ERK1/2 and PI3K/Akt pathways are mediated by EGF-induced EMT and cell migration and invasion in SBOT cells

**Figure 5:**
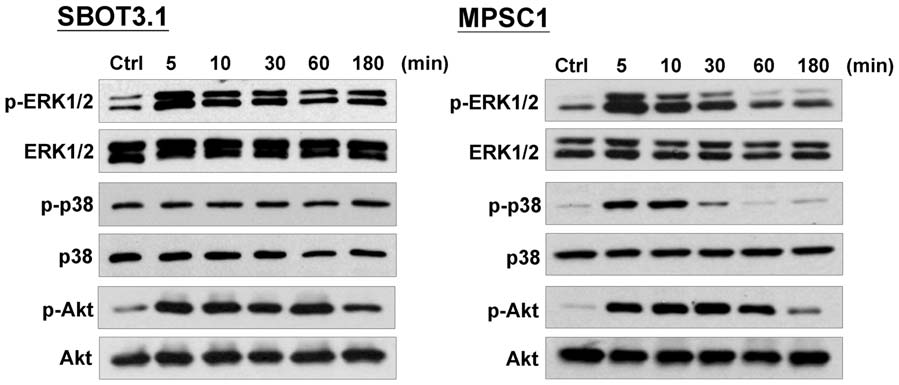
EGF activates ERK1/2 and Akt pathways in SBOT3.1 cells. SBOT3.1 and MPSC1 cells were treated with 50 ng/ml EGF for the indicated durations. Phosphorylation of ERK1/2, p38 MAPK and Akt were determined by western blot using antibodies specific for phosphorylated, activated forms of ERK1/2 (p-ERK1/2), p38 MAPK (p-p38) and Akt (p-Akt). Membranes were stripped and reprobed with antibodies to total ERK1/2, p38 MAPK and Akt.

**Figure 6:**
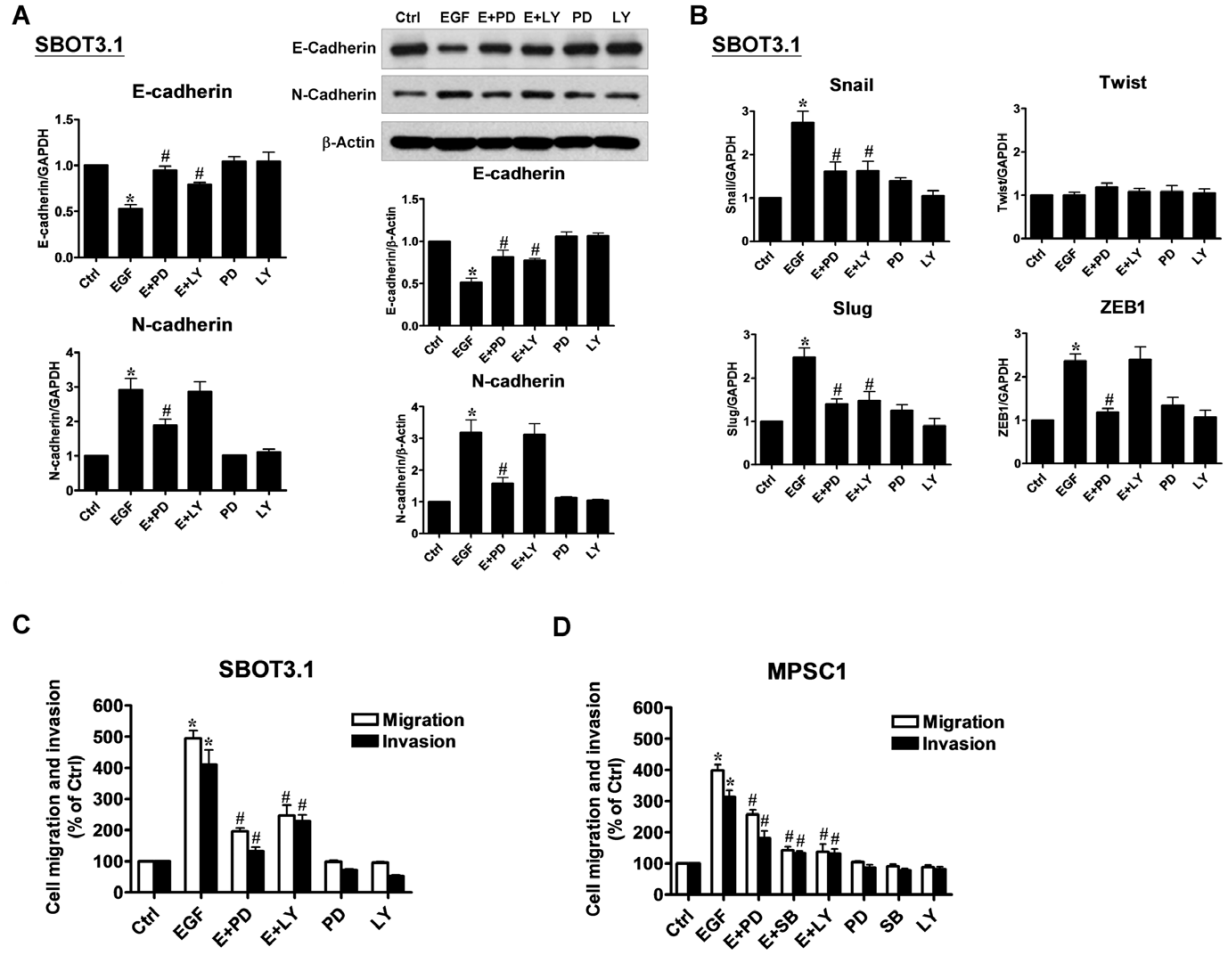
EGF induces cadherin switch through ERK1/2 and Akt activation in SBOT3.1 cells. A, SBOT3.1 cells were treated for 48 hr with PD98059 (20 µM) or LY294002 (20 µM) in the presence or absence of 50 ng/ml EGF. E-cadherin and N-cadherin mRNA (left panel) and protein (right panel) levels were analyzed by RT-qPCR and western blot, respectively. B, SBOT3.1 cells were treated for with PD98059 (20 µM) or LY294002 (20 µM) in the presence or absence of 50 ng/ml EGF and Snail, and the Slug, Twist and ZEB1 mRNA levels were analyzed by RT-qPCR. C, SBOT3.1 cells were treated with 50 ng/ml EGF in combination with PD98059 (20 µM) or LY294002 (20 µM). D, MPSC1 cells were treated with 50 ng/ml EGF in combination with PD98059 (20 µM) SB203580 (10 µM) or LY294002 (20 µM). After treatment, cells were seeded into un-coated (migration) and Matrigel-coated (invasion) transwell inserts. After 24 hr (migration) and 48 hr (invasion) incubation, non-invading cells were wiped from the upper side of the filter and the nuclei of invading cells were stained with Hoechst 33258. Results are expressed as the mean ± SEM of at least three independent experiments. *p<0.05 compared with Ctrl. ^#^p<0.05 compared with EGF.

It has been shown that the ERK1/2, p38 MAPK and PI3K/Akt pathways are involved in EGF-induced EMT [9], [22]. However, it is unknown whether these signaling pathways are also involved in EGF-induced EMT in SBOT cells. As shown in Figure 5, treatment with EGF induced the activation of ERK1/2 and Akt with the maximal effect observed at 5 min followed by a decrease after 180 min treatment. Interestingly, treatment with EGF did not activate p38 MAPK in SBOT3.1 cells. In contrast, EGF induced ERK1/2, p38 MAPK and Akt activation in MPSC1 cells. In SBOT3.1 cells, the EGF-induced down-regulation of E-cadherin and the up-regulation of N-cadherin mRNA and protein levels were diminished by treatment with the MEK1 inhibitor PD98059. Interestingly, treatment with the PI3K inhibitor LY294002 only diminished the EGF-induced down-regulation of E-cadherin but did not affect the EGF-induced up-regulation of N-cadherin (Figure 6A). In addition, treatment with PD98059 and LY294002 diminished EGF-induced up-regulation of Snail and Slug mRNA levels. However, the EGF-induced up-regulation of ZEB1 mRNA levels was only blocked by treatment with PD98059 and not with LY294002 (Figure 6B). Furthermore, EGF-induced cell migration and invasion were blocked by PD98059 and LY294002 treatments, although the inhibitory effect of LY294002 was less than that of PD98059 (Figure 6C). In MPSC1 cell, inhibition of ERK1/2, p38 MPAK and PI3K/Akt by PD98059, SB203580 and LY294002 attenuated EGF-induced cell migration and invasion (Figure 6D). Taken together, these results indicated that the ERK1/2 and PI3K/Akt pathways are involved in EGF-induced EMT and cell migration and invasion in SBOT cells. In addition, although EGF did not induce EMT in MPSC1 cells, our results indicate that ERK1/2, p38 MPAK and PI3K/Akt signaling pathways are involved in EGF-induced MPSC1 cell migration and invasion.

## Discussion

There is increasing evidence indicating that the activation of EGFR signaling contributes to cellular invasion in ovarian cancer by a variety of mechanisms. EGF treatment is known to increase cultured ovarian cancer cell migration, invasion, and proteolytic activity [23], [24]. Although the contributions of EGF and EGFR signaling have been described in ovarian cancer, the majority of studies have been performed only on high-grade ovarian cancer cells. In borderline tumors, immunohistochemical studies have shown that EGF and the EGFR are expressed, but there is no difference in EGFR staining intensity between benign, borderline and malignant ovarian tumors [25], [26]. Despite reports of EGFR expression in borderline tumors, the EGFR-mediated cell functions remain largely unknown. In the present study, we show that, consistent with previous immunohistochemical results, EGFR is expressed in cultured SBOT and LGC cells. It is well known that SV40 large T antigen (LT) inactivates p53 and retinoblastoma protein (Rb), whereas SV40 small T antigen (ST) inhibits the activity of the protein phosphatase 2A (PP2A) [27], [28]. It has been shown that the cell motility can be regulated by p53 and PP2A [28], [29]. In the present study, we used two SBOT cell lines which one is infected with SV40 LT (SBOT4-LT) and the other one is not (SBOT3.1). In addition, ILGC is the SV40 LT/ST immortalized LGC cell line, whereas MPSC1 is the LGC-derived cell line which does not carry SV40 LT/ST. Interestingly, although the four cell lines used in this study have different genetic backgrounds, our results show that treatment with EGF induced cell migration and invasion in all SBOT and LGC cell lines. These results suggest that p53/Rb and PP2A may not affect the EGF-induced cell migration and invasion in SBOT and LGC cells.

It has been shown that none of the EGF family of peptides can bind HER2, and this is important because HER2 is the preferred dimerization partner for all the other EGFR family members [5]. Overexpression of HER2 has been shown in high-grade ovarian cancer [30], [31]. However, other studies showed no relationship between HER2 expression and survival among patients with high-grade ovarian cancer [32], [33]. In SBOT and LGC, similar to high-grade ovarian cancer, HER2 expression and its association with prognosis are controversial [34], [35]. In the present study, we found that the expression levels of HER2 were similar in two SBOT and two LGC cell lines. However, whether HER2 is involved in EGF-induced SBOT and LGC cell motility remains unknown.

In ovarian cancer, based on molecular genetic and morphological studies, it has been suggested that there are two pathways of tumorigenesis that correspond to the development of low-grade and high-grade serous ovarian carcinoma [36]. In type I tumors, invasive LGC develops from a non-invasive SBOT. Histopathologic and molecular genetic studies suggest that SBOT may arise from ovarian surface epithelium (OSE) or cystadenomas [37]. In humans, OSE has either a flat or a cuboidal appearance. Flat OSE does not express E-cadherin. In the ovary, E-cadherin expression is limited to rare regions such as cuboidal and columnar OSE, where cells resemble metaplastic epithelium [38], [39]. Immunohistochemical studies showed that membranous E-cadherin expression is detected in benign and serous borderline ovarian tumors. Importantly, reduced expression of E-cadherin correlates with the presence of microinvasion in serous borderline tumors [40]. Our recent study in cultured SBOT cells also showed that down-regulation of E-cadherin contributes to the progression of SBOT to invasive LGC [16]. Taken together, these results suggest that the expression of E-cadherin occurs intermittently during the progression from OSE to SBOT to invasive LGC and may be required for the initiation of tumorigenesis in type I tumors. Therefore, we hypothesize that once normal OSE acquires the expression of E-cadherin, which may play a role in early events leading to the malignant phenotype, the subsequent EMT may be required for the progression of a non-invasive tumor to an invasive tumor.

Although the key feature of EMT is the down-regulation of E-cadherin and up-regulation of N-cadherin, there still are some other molecular markers that are used for EMT, such as increased expression of vimentin, fibronectin and nuclear localization of β-catenin and decreased expression of the tight junction protein, occluding [2]. However, the transition from epithelial to mesenchymal cell characteristics encompasses a spectrum of inter- and intracellular changes, not all of which are always seen during EMT [41]. In the present study, we show that EGF treatment induced a switch from E-cadherin to N-cadherin expression in SBOT cells. However, the effect of EGF on other EMT markers requires further investigation. Here, we show that EGF treatment down-regulates E-cadherin expression in SBOT cells. In contrast, no such changes were observed in LGC cells. The western blot results show that the EGFR level was higher in SBOT3.1 cells than in MPSC1 cells, indicating that the effects of EGF on cadherin switch are not related to the levels of EGFR. A recent study showed that different binding affinities between EGF and EGFR activate different signaling pathways. High-affinity EGF binding is sufficient for activation of most canonical signaling pathways, whereas low-affinity EGF binding is required for the activation of the STATs and PLCγ1 [42]. Many signaling pathways have been reported to be involved in the EMT in ovarian cancer [43]. It will require further investigation to examine whether the divergent effects of EGF on the cadherin switch result from the different binding affinities between EGF and EGFR in SBOT and LGC cells. In high-grade ovarian cancer cells, we recently showed that H_2_O_2_ mediates the EGF-induced down-regulation of E-cadherin expression in SKOV3 ovarian cancer cells and suggested that the lack of an effect of EGF on E-cadherin in OVCAR3 cells may reflect an uncoupling of EGFR activation from H_2_O_2_ production [22]. However, because the EGFR is functional, as shown by detection of activated EGF-induced EGFR phosphorylation, ERK1/2, p38 MAPK and PI3K/Akt, it is unclear whether the lack of an effect of EGF on E-cadherin expression in MPSC1 cells is due to the lack of H_2_O_2_ production after EGF treatment.

Reduced expression of E-cadherin in human cancers is associated with metastasis, whereas in high-grade ovarian cancer, forced expression of E-cadherin inhibits tumor metastasis [44]. We have shown that endogenous E-cadherin plays an important regulatory role in cell invasion and that EGF-induced cell invasion is mediated by the down-regulation of E-cadherin expression in high-grade ovarian cancer cells [22]. In SBOT cells, our recent study showed that the down-regulation of E-cadherin by the PI3K/Akt pathway contributes to the progression to the invasive phenotype [16]. In this study, we show that LGC-derived MPSC1 cells express lower levels of E-cadherin and higher levels of N-cadherin than SBOT cells, suggesting that EMT may contribute to the progression from SBOT to invasive LGC.

In the present study, our data demonstrate that in SBOT cells, ERK1/2 and Akt mediated the EGF-induced down-regulation of E-cadherin expression, whereas only ERK1/2 was involved in EGF-induced N-cadherin expression. Down-regulation of E-cadherin is mainly due to the up-regulation of Snail, Slug, Twist, ZEB1 and other transcription factors that repress E-cadherin [3]. We show here that the expression of Snail, Slug and ZEB1, but not Twist, was increased by EGF treatment in SBOT cells. Recent studies have shown that Twist and ZEB1 not only repress E-cadherin expression but also induce the expression of N-cadherin [45], [46]. Treatment with LY294002 did not block the EGF-induced up-regulation of N-cadherin, which may be due to the lack of an inhibitory effect of LY294002 on ZEB1 expression. Nevertheless, both the ERK1/2 and PI3K/Akt pathways were involved in EGF-induced SBOT cell migration and invasion. These results are consistent with our previous finding that E-cadherin, but not N-cadherin, plays an important role in the regulation of SBOT cell invasion [16], [17], [18].

In summary, this study demonstrates that EGFR is expressed in cultured SBOT and LGC cells and that treatment with EGF induces cell migration and invasion by activating EMT in SBOT cells, which may play an important role in the progression from SBOT to invasive LGC. In addition, this study suggests that there may be E-cadherin-independent mechanisms that mediate the EGF-induced cell migration and invasion in LGC cells.
